# Review of the 2023 JVDI corresponding author survey

**DOI:** 10.1177/10406387231176225

**Published:** 2023-05-19

**Authors:** Grant Maxie

JVDI Editors and the AAVLD Executive Board were eager to get feedback on authors’ satisfaction with the JVDI editorial process. Manuscript submissions have declined somewhat from a peak in 2020 (COVID pandemic related?), as has occurred across the board for Sage and other journals, and we wanted to investigate possible reasons for the decline. Hence, in April 2023, we sent a SurveyMonkey questionnaire to the corresponding authors of articles published in JVDI in 2021 or 2022. We received 53 responses to the surveys sent to 265 JVDI corresponding authors. Here is a summary of responses:

*Q1. Why did you select JVDI for your article?*

All 53 respondents commented. Most noted that the scope of the journal was appropriate to their topic, that their target audience would be reached, that JVDI has a good reputation, or that the journal’s impact was suitable. Six noted specifically that JVDI publishes case reports.

*Q2. Were JVDI Instructions for authors easy to follow?*

Very satisfied (23), satisfied (26), neutral (4), dissatisfied (0).

*Q3. Was submission through SAGE Track efficient?*

Very satisfied (26), satisfied (26), neutral (1), dissatisfied (0).

*Q4. Was the time to first decision for your submission satisfactory?*

Very satisfied (16), satisfied (24), neutral (10), dissatisfied (1), very dissatisfied (2).

*Q5. Were your images handled to your satisfaction?*

Very satisfied (26), satisfied (24), neutral (2), dissatisfied (1), very dissatisfied (0).

*Q6. What did you like most about submitting to JVDI?*

Of the 50 comments received, most reflected positively on the rigor of the peer-review process, constructive comments from reviewers and editors, and the interactions with reviewers and editors.

*Q7. Would anything in your interaction with JVDI prevent you from submitting to JVDI again?*

Most respondents (39 of 51) had no reservations about submitting again; 12 of 51 had reservations based on longer-than-expected turnaround times or unsatisfactory interactions with reviewers or editors; 3 would likely not submit again. Survey responses were anonymous; hence follow-up on specific concerns is not possible. I’m happy to receive author feedback, both positive and negative, on individual submissions.

*Q8. What is your overall level of satisfaction with publishing in JVDI?*

Very satisfied (26), satisfied (21), neutral (1), dissatisfied (3), very dissatisfied (1).

*Q9. How many other peer-reviewed publications have you authored or co-authored in the past 2 years?*

Range of 0–35 publications from the 53 respondents; mean of 7 other publications in 2021–2022; published in > 90 different journals (some answers were a count of journals only).

*Q10. Other comments to the JVDI Editorial Board for improving the JVDI publishing experience are welcome.*

**Figure 1.**
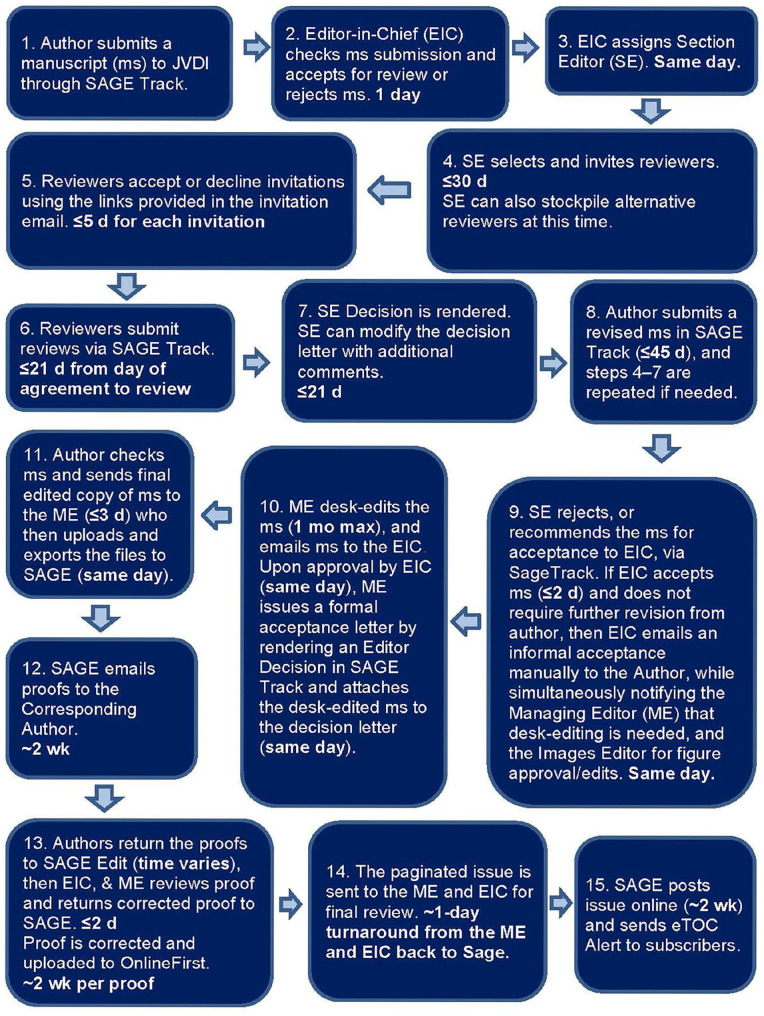
Flowchart of the JVDI editorial process.

Of the 24 comments, most were complimentary or even laudatory of the editorial process; one author was very dissatisfied with the editor’s comments. One author noted an inconsistency between our Instructions and our Best Practices PowerPoint—we have revised these documents accordingly. One author would like speedier editing and final publication timing. We continue to review the elapsed times in the various steps of our review, editing, and publication process, and we have tightened them as much as possible (Fig. 1).

Soliciting peer reviews can be arduous. My record to date is of 18 potential reviewers declining, or being unavailable, to review! Inviting alternative reviewers then prolongs the elapsed review time. Delays in finding willing reviewers are inescapable. We try to reward reviewers by copying the other reviewer’s comments to them, sharing the responses of authors and editors with reviewers, and publishing an annual thank you to reviewers. Sage offers reviewers 60-d complimentary online access to all journals published by Sage, as well as a 25% discount on all Sage books ordered online. Sage also collaborates with Publons to give reviewers recognition for their peer review contributions. Once in the hands of Sage, accepted articles are published quickly online and abstracts are available to all; once compiled into an issue of the *Journal*, a full-issue cover-to-cover PDF is then freely available to AAVLD members.

As noted in our 2017 AAVLD communications survey,^1^ 78% of our readers were satisfied overall with JVDI and found our content relevant to current events, to their jobs, and to continuing education. There was continued strong support for publication of case reports, as also noted in our 2022 readership survey,^2^ which meshes with the 2021–2022 submissions from corresponding authors. As always, readers (who are often authors) would like to see a faster review process and prompter publication, but most recognize that editors and reviewers are volunteers with competing priorities; we do our best to enforce deadlines.

We will continue to update our *Instructions for authors* and *Best practices for submitting, reviewing, and publishing manuscripts in JVDI* (located at https://journals.sagepub.com/author-instructions/VDI and in the Author Center at https://mc.manuscriptcentral.com/jvdi) to facilitate trouble-free production and submission of manuscripts.


Grant Maxie, DVM, PhD, DACVP *Editor-in-Chief*

